# Recent trends in tuberculosis, Japan.

**DOI:** 10.3201/eid0606.000602

**Published:** 2000

**Authors:** T Mori

**Affiliations:** Research Institute of Tuberculosis and Anti-Tuberculosis Association, Japan. tmori@jata.or.jp

## Abstract

Despite a decline after World War II, the rate of tuberculosis in Japan remains high. Infection is heavily concentrated in the > or =60-year age group, and 82% of patients are > or =40 years of age. The success rate for treatment of smear-positive patients is 78%. Multidrug-resistant strains of Mycobacterium tuberculosis are rare.


### Recent Trends in Tuberculosis, Japan

**Toru Mori**
Research Institute of Tuberculosis, Japan
Anti-Tuberculosis Association, Tokyo, Japan

Despite a decline after World War II, the rate of tuberculosis in Japan remains high. Infection is heavily concentrated in the ≥60-year age group, and 82% of patients are ≥40 years of age. The success rate for treatment of smear-positive patients is 78%. Multidrug-resistant strains of *Mycobacterium tuberculosis* are rare.

Before World War II, tuberculosis (TB) was highly prevalent in Japan. After the war, TB control measures came into widespread use, and both the incidence and death rate decreased rapidly, as in the United States and other industrialized nations (Figure 1). Because of the high rate in the postwar period, however, the TB case rate remains higher in Japan than in other countries. In 1998, the incidence of all forms of TB was 34.8 per 100,000 population in Japan, compared with 6.4 in the United States, 9.5 in the Netherlands, and 4.7 in Norway (the data from Norway are for 1997). The annual risk for infection was estimated to be 4% in the 1950s and 0.05% in the 1990s, reflecting an 11% annual rate of decrease from the 1950s to the 1970s, with a less steep decline thereafter, in parallel with the decrease in death and case rates.

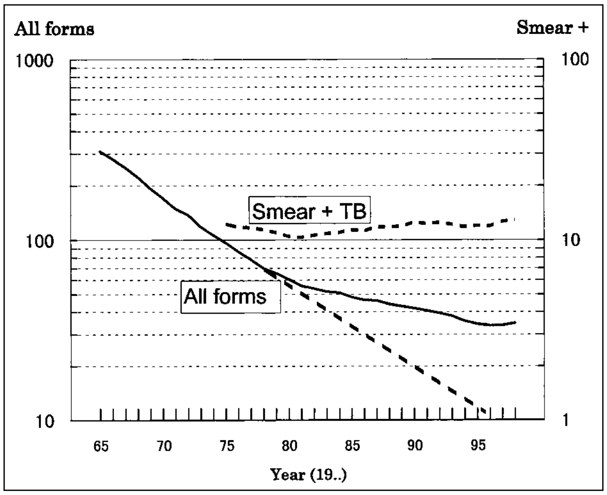

Figure 1. Tuberculosis (rate per 100,000 population) in Japan, 1965-1995.

Address for correspondence: Toru Mori, Research Institute of Tuberculosis, Kiyose, Tokyo 203-8835, Japan; fax: +81-424-92-4600; e-mail: tmori@jata.or.jp.

### Epidemiologic Characteristics

Epidemiologic data show that after years of continuous decline, TB incidence is changing in Japan. In 1950, more than half of adolescents ages ≤20 years are estimated to have been infected, which resulted in a peak of TB incidence and deaths in this age group (Figure 2). In 1995, 1% of those ages ≤20 years and 2% of those aged ≤30 years were infected. A high TB rate among persons ages ≥60 years reflects infection acquired during youth.

Along with the shift in age-specific prevalence of infection, aging of the population during this period contributes to the disproportionate TB rate in older age groups. The proportion of newly reported patients ages ≥40 years was 53% in 1950 and 82% in 1998. These data indicate that the elderly, as well as the physically and socioeconomically disadvantaged, are at high risk for TB.

HIV infection, an important risk factor for TB worldwide, has not been widespread in Japan. However, a voluntary reporting system for TB

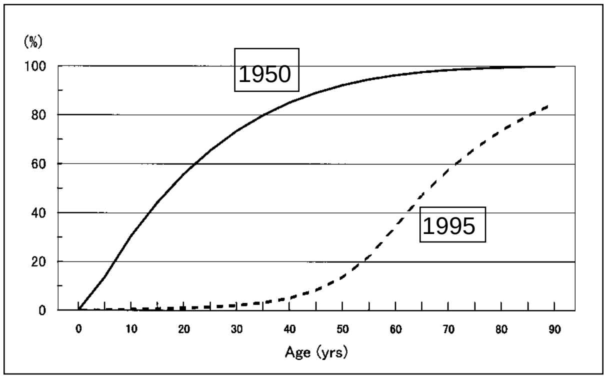

Figure 2. Estimated prevalence of tuberculosis infection by age in Japan, 1950 and 1995.

cases in HIV-infected persons, involving 14 physicians throughout the country, indicates that 90 such cases had been reported as of 1998 and that this number is increasing.

As in the United States, TB is becoming more common in economically disadvantaged households and workers in certain small businesses. The case rate among the disadvantaged and homeless in metropolitan areas is 50 to 60 times higher than the rate among the general population. TB among immigrants has been a problem in Japan, but in 1996 the proportion of TB patients who had entered Japan within the previous 5 years was only 1% of all newly reported cases.

### Clinical Characteristics

The proportion of TB cases that are bacteriologically confirmed among all newly reported pulmonary cases has been increasing, from 19% in 1975 to 51% in 1998. Part of this change can be attributed to reporting that favors bacteriologic evidence over X-ray findings. However, data confirm that cases with severe symptoms or extensive disease at onset have increased. The number of patients with newly reported cases who die within a year of reporting has slowly increased, from 1.6% in 1975 to 2.6% in 1997.

According to a nationwide survey of TB treatment programs conducted in 1995, among smear-positive patients, 78% were cured, 12% died within 9 months, 7% were lost to follow up, and 3% did not respond to treatment. Throughout Japan, drug resistance has been generally low, as shown by a nationwide survey conducted at 5-year intervals (Table). Resistance to both isoniazid and rifampicin among previously untreated patients, i.e., multidrug resistance, is still low.

Table. Percentage of drug resistance in tuberculosis patients without prior treatment, Japan, 1997

| Drug | Frequency (95% CI) |
|---|---|
| Isoniazid | 4.4 (3.4-5.7) |
| Rifampicin | 1.4 (0.8-2.2) |
| Streptomycin | 7.5 (6.2-9.0) |
| Ethambutol | 0.4 (0.2-0.9) |
| Any drug | 10.2 (8.7-11.9) |
| Resistant to both Isoniazid and Rifampicin | 0.8 (0.4-1.4) |

CI = confidence intervals

### Recent Slowing of Decline in TB Cases

The rate of decline in TB cases of all forms has slowed from 11% to 3% since 1980, and after 1996, the trend reversed slightly. The case rates for 1996, 1997, and 1998 were 33.7, 33.9, and 34.8, respectively. The reversal was most obvious in persons $\geq$70 years of age, but the plateau after 1980 is seen in all age groups, including adolescents and children $\leq$14 years of age. For smear-positive pulmonary TB, the downward trend reversed earlier, especially among the elderly. This reversal may be attributable to the rapid growth of the older age groups, who have high rates of past TB infection and are affected by conditions (e.g., diabetes, corticosteroid therapy, or hemodialysis) that increase their risk for TB. Patients in this older age group are a source of infection, transmitting TB to younger generations and leading to the plateau in case rates.

Another consequence of epidemiologic changes is an increase in small outbreaks of TB. While outbreaks used to be seen primarily in schools, now they are also seen in settings frequented by the young, including places of work and entertainment (e.g., bars, karaoke houses, public saunas). The main epidemiologic basis for the increase in outbreaks is the gap in infection prevalence between age groups, e.g., young, susceptible persons living in close contact with the elderly, who are vulnerable to TB infection (Figure 2).

Nosocomial outbreaks of TB are also a problem. Nationwide surveillance data show that during 1987 through 1997, the case rate of all forms of TB among female nurses was 2.3 times higher than that for the general female population. The relative risk among nurses was highest at 3.3 in the 20- to 29-year-old age group; risk declined with age but is still substantially higher for those <60 years of age.

### Declaration of National TB Emergency

Control programs supported by government and the medical profession influence TB trends in Japan. An indicator of TB awareness is physician delay in case detection, i.e., the time from a patient's first visit until diagnosis of TB. According to national surveillance, 62% of all pulmonary cases are detected within 1 month of the first visit, but 15% of cases are detected after 2 months of medical attention. This interval before diagnosis is expected to increase when TB becomes less frequent, with an accompanying decline in physician awareness of the disease. Because of the recent increase in TB rates, the Japanese minister of health and welfare has declared a TB emergency, urging the public, as well as the medical profession and local governments, to be on the alert for TB so further increases can be prevented.

